# Enantioselective Mixed Matrix Membranes for Chiral Resolution

**DOI:** 10.3390/membranes11040279

**Published:** 2021-04-10

**Authors:** Hwa-Jin Choi, Yun-Ho Ahn, Dong-Yeun Koh

**Affiliations:** 1 Department of Chemical and Molecular Engineering (BK-21 Plus), Korea Advanced Institute of Science and Technology, Daejeon 34141, Korea; hwajinchoi@kaist.ac.kr; 2 Department of Chemical Engineering, Soongsil University, 369 Sangdo-ro, Dongjak-gu, Seoul 06978, Korea; yhahn@ssu.ac.kr

**Keywords:** chiral resolution, enantioselective process, racemic mixture, diffusion, molecular interaction

## Abstract

Most pharmaceuticals are stereoisomers that each enantiomer shows dramatically different biological activity. Therefore, the production of optically pure chemicals through sustainable and energy-efficient technology is one of the main objectives in the pharmaceutical industry. Membrane-based separation is a continuous process performed on a large scale that uses far less energy than the conventional thermal separation process. Enantioselective polymer membranes have been developed for chiral resolution of pharmaceuticals; however, it is difficult to generate sufficient enantiomeric excess (ee) with conventional polymers. This article describes a chiral resolution strategy using a composite structure of mixed matrix membrane that employs chiral fillers. We discuss several enantioselective fillers, including metal-organic frameworks (MOFs), covalent organic frameworks (COFs), zeolites, porous organic cages (POCs), and their potential use as chiral fillers in mixed matrix membranes. State-of-the-art enantioselective mixed matrix membranes (MMMs) and the future design consideration for highly efficient enantioselective MMMs are discussed.

## 1. Introduction

In the pharmaceutical and food industries, many enzymatic reactions or other metabolic activities rely on a specific enantiomer of a chiral compound, and high enantioselectivity is essential to obtain the targeted pharmacological effect [1]. The use of ‘asymmetric synthesis’ can produce a single type of enantiomer from an achiral source. Several synthetic methodologies have been developed to achieve enantiomerically enriched products (e.g., amino acids, pharmaceuticals), including asymmetrically designed enzymes [2], the asymmetric activation of enantiomeric catalysts [3], and catalysts with chiral hydrogen-bond-donor ligands [4]. Researchers have tried to find milder reaction conditions, less expensive commodity chemicals, and strategies to enhance the stability of catalysts to develop commercially reliable and large-scale asymmetric synthesis processes [2,3,4]. However, since expensive raw materials are still required in the available asymmetric synthesis processes, large-scale production has rarely been reported. Instead of producing homochiral species, separation of the enantiomers (i.e., optical resolution or chiral separation) has become an important technique and should be investigated further for various practical applications. 

In the last few decades, four conventional chiral resolution methods have been developed: crystallization, kinetic separation, chromatography, and membrane-based separation. Pasteur firstly developed crystallization-based separation of tartaric acid in 1848, a straightforward approach that involves a simple, low-cost chemical reaction [5]. However, separating two different crystals at an industrial scale makes it challenging to develop an economically feasible chiral resolution process via crystallization [6]. A prerequisite for employing kinetic separation is that the two enantiomers should react with a chiral entity at different rates. Since kinetic separation is based on a catalytic reaction, decreasing catalytic activity over time is also a critical issue affecting application at an industrial scale [7,8]. Chromatography is a widely used and successful technique in the chiral resolution field. The enantioselective binding affinity of the chiral stationary phase in chromatography enables selective sorption of one type of enantiomer; thus, one enantiomer remains longer in the column while the other enantiomer passes through the column. Polysaccharides are representative chiral polymers that have been commercially utilized in high performance liquid chromatography (HPLC) with high enantiomeric excess (ee) values [9,10,11,12]. Since chromatography has been highly effective at the analytical or lab-scale, the concept of simulated moving bed (SMB) chromatography for chiral resolution was proposed for practical applications [13].

Although crystallization, kinetic separation, and chromatography have been extensively investigated, the existing chiral resolution methods still utilize batch technology. The shift towards continuous manufacturing in the pharmaceutical and food industries now provides an opportunity for steady-state, enantioselective membrane-based separations to emerge as a platform technology for large-scale chiral resolution. Membrane-based separation generally has distinct advantages, such as the low energy consumption of the process [14], solution processability of material [15], the large specific surface area of membrane module [16], and a tunable pore structure of membrane materials [17]. Recently, organic solvent nanofiltration (OSN, in other words, solvent-resistant nanofiltration) has emerged as a new low–energy and low–carbon technology to improve the sustainability of the conventional solvent separation processes. Therefore, membranes are a promising platform for chiral resolution associated with various types of solvents [16]. Since membrane-based separation can be performed continuously in a single unit, a large amount of enantiomeric mixture can be separated efficiently at an industrial scale. 

The membrane-based separation of D–/L–tyrosine or tryptophan was firstly reported in 1990, and the membrane was fabricated by coating poly(L–glutamates) on ultrafiltration membrane supports [18]. Many advances in membrane–based chiral resolution have been reported since this work. Various strategies have been suggested for producing chiral resolution membranes with high enantioselectivities, including using chiral polymers [18,19,20,21,22,23,24,25,26] or chiral microporous materials [27,28,29,30,31]. In this review, we highlight the membrane-based approach in enantioselective separation processes. Among various types of membranes, we focus on the enantioselective mixed matrix membrane (MMM), which possesses the advantages of both a solution-processable polymeric matrix and a highly enantioselective microporous filler. We discuss several enantioselective microporous materials, including metal-organic frameworks (MOFs), covalent organic frameworks (COFs), zeolites, porous organic cages (POCs), and their potential use as chiral fillers in MMMs. Additionally, we introduce the fabrication methods and enantiomeric separation performances of state-of-the-art enantioselective MMMs. Future design considerations for the enantioselective MMMs are suggested in the last section to implement highly efficient, large-scale enantiomeric separations.

## 2. Towards A Membrane-Based Chiral Resolution

### 2.1. Chiral Resolution via Mixed Matrix Membranes (MMMs)

Various microporous polymers have been investigated for the scalable separation of gas or solvent-solute pairs based on the solution–diffusion mechanism [32]. Solution processability enables the continuous production of polymeric membranes in various forms (e.g., films, hollow fibers) at large scales. However, polymeric membranes are typically unstable in organic solvents and exhibit low permeation flux or separation capacity [33]. Microporous materials, including metal-organic frameworks (MOFs), covalent organic frameworks (COFs), zeolites, and porous organic cages (POCs), have been broadly recognized as promising alternatives due to their high porosity and adjustable pore structure. However, preparing membranes with these microporous materials on porous supports is relatively difficult due to the complex interactions (e.g., nucleation, intergrowth, etc.) between the crystals and the support materials. Besides, the formation of defects or pinholes is inevitable during the three-dimensional growth of microporous membranes on the support materials. 

Alternatively, mixed matrix membranes (MMMs) combine the advantages of polymeric membranes and microporous materials: microporous materials with high separation performance can be easily dispersed as ‘filler’ particles in the polymeric matrix via solution processes. MMMs have already shown improved separation performance for solvent separations [34] and gas separations [35]. For example, an MMM which contained discrete fillers of HKUST-1 in a continuous polyimide P84 phase showed higher rejections of the polystyrene markers than pure P84 ultrafiltration membranes [36]. The organic solvent nanofiltration (OSN) performance of MMM can be improved via further modifications, such as the in situ growth of HKUST–1 within the pores of the P84 membrane, or by introducing carboxylate functional groups to increase the degree of adhesion of HKUST–1 to the membranes, which achieved a molecular weight cutoff of 794 g/mol [37].

A Robeson’s upper bound exists for gas separation membranes, representing the trade-off relationship between selectivity and permeability. Although many researchers have strived to optimize the membrane fabrication condition or develop new polymeric materials to improve the separation performance of polymeric membranes, conventional membranes generally cannot exceed Robeson’s upper bound. However, the upper bound can often be overcome using inorganic membranes fabricated with microporous materials, such as carbon molecular sieve (CMS) and zeolite [38,39]. These microporous materials can also be incorporated into the polymeric matrix to form MMMs that enhance the separation performance of the polymeric membrane. The size- and shape-selective nature of microporous materials allows molecular sieving with different diffusion rates: smaller molecules diffuse faster than larger molecules. The confined, limited space of micropores restricts the free motion of gas molecules, resulting in a higher entropic selectivity, which enables more delicate separation than conventional polymeric materials [39]. However, simple molecular sieving cannot be used in enantiomeric separations. Two enantiomers cannot be separated through the same molecular sieve membranes since the sizes of the two enantiomers are identical to each other. The enantioselective MMMs must have chiral recognition sites to separate enantiomers, and incorporating chiral fillers can induce this chirality in the membrane.

### 2.2. Transport Mechanisms in Enantioselective Membranes

**Figure 1:**
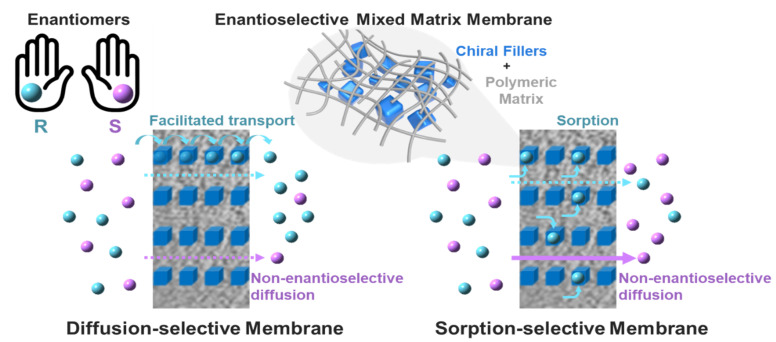
Schematic diagram of principles between a diffusion-–elective membrane and sorption–selective membrane.

There are two suggested mechanisms for chiral resolution via membrane-based process: facilitated transport and retarded transport. Generally, enantioselective membranes can be divided into two classes: ‘diffusion-selective’ membranes and ‘sorption-selective’ membranes, which employ facilitated and retarded transport mechanisms, respectively [16,40,41,42] (Figure 1). In a diffusion-selective membrane, one type of enantiomer with a higher binding affinity can dominantly interact with the chiral active sites in the membrane. In this way, preferential adsorption from the feed stream and the rapid transfer from one to another chiral site can be ‘facilitated’ under a chemical potential gradient. The highest ee value (~100%) can be obtained by selective permeation of the enantiomer in the initial period of separation. However, the resolution performance decreases over time due to the non-enantioselective diffusion of another type of enantiomer. 

Many enantioselective membranes composed of chiral polymers or chiral microporous materials have shown diffusion-selective behaviors [16,40,41]. In a sorption-selective membrane, the binding affinity between one type of enantiomer and a chiral recognition site in the enantioselective membrane is stronger than in a diffusion-selective membrane: the adsorbed single enantiomer can be retained in the membrane. As a result, the non-selective enantiomer dominantly diffuses through the membrane under a chemical potential gradient. However, although sorption-selective membranes have rarely been reported, two enantioselective mixed matrix membranes composed of modified single-walled carbon nanotubes and β–cyclodextrin showed retarded transport behavior (to be discussed in Section 4) [43,44]. Interestingly, MD simulation predicted that in a graphene-based membrane, the transport mechanism could be converted from a facilitated mechanism to the retarded mechanism or vice versa depending on the separation environment (e.g., the interlayer distance of the two-dimensional materials) [45]. Thus, understanding the transport mechanism occurring in the enantioselective membrane is essential for designing membrane-based chiral resolution.

## 3. Mixed Matrix Membranes in Various Combinations

### 3.1. Chiral Filler: Metal-Organic Frameworks (MOFs)

MOFs are an emerging class of porous materials showing structural and textural diversity. Their pore sizes can be adjusted by combining various types of ligands and center metals to allow the separation of a wide variety of molecular mixtures [46]. MOFs also enable the delicate separation of gas/organic molecules of almost equal sizes through the molecular sieving mechanism. Chiral MOFs have also garnered tremendous attention because of their potential for enantiomer separation, although, as noted, the enantiomeric separation cannot be achieved via simple molecular sieving. Instead, the pores can be tuned to provide chiral environments for enantiomeric mixtures [47]. A chiral MOF, (R)-CuMOF–1–silica composite, has a strong binding affinity to a single type of enantiomer and was utilized as a chiral stationary phase in the high-performance liquid chromatography (HPLC) to separate enantiomeric mixtures including racemic sulfoxides, *sec*-alcohols, *β*–lactams, benzoins, and flavanones epoxides [48]. A chiral stationary phase in the HPLC column must meet the application-specific resolution of the enantiomers, and some reported chiral MOFs are plausible candidates [49,50,51,52]. 

An enantiomeric mixture can be separated based on the difference in interaction with the ligand within the MOF structures. Kuang et al. synthesized enantioselective Zn–based MOF, [ZnLBr]·H_2_O (L: N–(4–Pyridylmethyl)–L/D–leucine·HBr), which has the proper pore size and helical channel to separate (±)-ibuprofen, (±)-phenylethylamine and (±)-1-Phenyl-1-propanol [53]. In general, helical structures can be constructed using both chiral and achiral sources. Utilizing chiral sources to design helical complexes can result in inherent chirality. Also, symmetry breaking with an achiral source can produce a helical structure [54]. [ZnLBr] was synthesized using a homochiral precursor, *N*–(4–Pyridylmethyl)-*L*-leucine·HBr, the aperture size was calculated around 9.8 A°. (±)–ibuprofen was effectively separated, demonstrating chromatographic resolution (Rs = 4.1) [53]. Another type of MOF constructed with long-chain chiral ligands was reported: (R)-CuMOF–2, which enabled the efficient separation of enantiomeric mixtures such as 3-phenoxy–1,2–propanediol, styrene oxide, phenyl glycidyl ether, and γ–phenyl–γ–butyrolactone [55]. 

Besides the molecular interactions with MOFs, hydrogen bonding, π−π stacking, hydrophobic interaction, van der Waals forces, and dipole−dipole interactions can affect enantiomeric excess. Among those interactions, some researchers have highlighted the hydrogen bonding between the ligand of chiral MOFs and the functional group of enantiomers, noting that van der Waals and π···π interactions are the critical factors that determine the separation efficiency [56,57]. Since enantiomers have a different stereo configuration, their interaction with the ligand might be different. In biphenol-based MOFs, two types of chiral ligands offer distinct orientations to the target enantiomers. The specific binding energies in the chiral channel of the framework can affect the enantioselectivity [49]. Thus, the size of the chiral channels in enantioselective MOFs and molecular interaction can play a part in chiral recognition [50,53,55]. For example, (±)–1–phenyl–1–propanol with a hydroxyl group and (±)–1–phenylethylamine with an amino group showed different behaviors in the chiral channels of Zn-based MOF ([ZnLBr]·H_2_O) [53]. (+)–1–phenylethylamine was retained longer due to a stronger interaction with the framework. These observations proved that molecules with a specific size could easily pass through the chiral channel and interact with the chiral framework [53]. Furthermore, the enantioselectivity of chiral MOFs can be affected by various factors such as the geometry of crystals, temperature, substitution, and acidity. Wu et al. discovered that pH value determines the shape of the MOF crystal, and different corresponding ee values can be obtained [58]. Peng et al. observed *p*–substituted 1–phenylethylamine (1–PEA) exhibited high enantioselectivity, whereas *o*–and *m*-substituted 1–PEA showed ordinary enantioselectivity [49]. 

**Figure 2:**
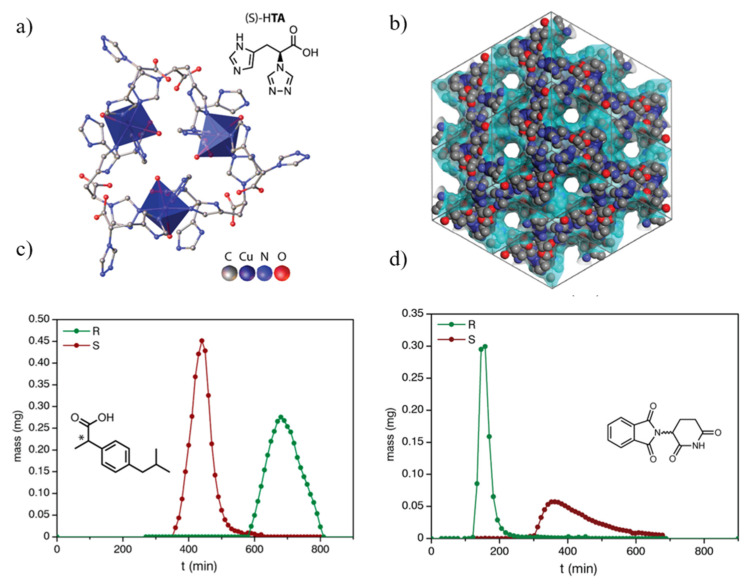
(**a**) Structure of TAMOF–1 and the linker L–2–deaza–2–(4H–1,2,4–triazol–4–yl)histidine (S–HTA). (**b**) Crystal structure on (111) plane, which indicates intersected helicoidal channels (**c**). (**d**) Gravity chromatographic separations of (±)-ibuprofen and (±)–thalidomide, respectively. Reproduced with permission from [51] Copyright 2019 American Chemical Society.

The chiral MOFs used to fabricate MMMs should be carefully chosen with consideration of several characteristics. The scalability and stability of chiral MOF are critical factors. TAMOF–1 is a representative chiral MOF with high enantioselectivity that can be synthesized at a large scale [51]. TAMOF–1 was adopted for the chiral stationary phase of an HPLC. A packed column of TAMOF–1 successfully separated (±)–ibuprofen and (±)–thalidomide with different retention times (Figure 2). TAMOF-1 also has a mechanically stable structure, resulting from its strong metal–nitrogen bonds, providing superior chemical stability. Since TAMOF-1 has a high chemical stability and ee value, it is expected that TAMOF–1 would be an excellent candidate for fabricating MMM with a proper polymer matrix and retain its high enantioselectivity for steady-state operation [51]. Considering the solution process of MMM fabrication, the chemical stability of the chiral MOF to a solvent is another essential factor.

The microporous solid fillers are dispersed in a solution state and converted into a solid form of membrane film or fiber. The chiral MOF crystals need to remain intact in a solvent/nonsolvent mixture or polymer solution during the entire enantioselective MMM fabrication process. For example, enantioselective MOFs with excellent chemical stability have been reported: HMOF–1, in particular, can maintain its two-fold DNA–like helical structure under harsh solvent conditions, including acidic and basic solutions [59].

#### Dispersed Chiral MOF Fillers in an Achiral Polymeric Matrix

State-of-the-art studies on enantioselective MMMs have focused on the use of chiral MOF fillers dispersed in the achiral polymeric matrix (Table 1). Chirality-induced MOFs ((M)–Eu(BTC) or (P)–Eu(BTC)), which were synthesized by adding a chiral dopant ((R)–(–)–2–Amino–1–butanol or (S)–(+)–2–Amino–1–butanol), have been utilized as chiral fillers for mixed matrix membranes [60]. Polished porous silica discs were coated with a MOF filler-dispersed polymer solution ((M)-Eu(BTC) or (P)–Eu(BTC) + achiral PIM–1 + CHCl_3_). The coated discs were dried slowly at room temperature, and these coating and drying procedures were repeated a couple of times to fabricate the enantioselective Eu(BTC)/PIM–1 mixed matrix membrane. The optimized amount of fillers in this MMM was 30 wt%. As this MMM was composed of a PIM–1 material, the type of solvent could affect the enantioselective performance: polar solvents (water, ethanol) lower the *ee* value of MMM more than a non-polar solvent (*n*-hexane). The highest ee value of 9% was observed for 2-amino-1-butanol. This enantiomer separation performance can be improved via the further increase in chiral MOFs’ enantiopurity or selection of different solvents. In fact, the enantiomer separation performance can be significantly enhanced using homochiral fillers. A homochiral MOF (Zn–BLD) was prepared from the chiral linker of *L*-Lactic acid, and Zn–BLD nanocrystals were dispersed in the melt of high–density polyethylene (HDPE) and paraffin at 200 °C [61]. The Zn–BLD/HDPE mixed matrix membrane was fabricated via roll–to–roll hot pressing at 120 °C, and this MMM could retain 86 wt% of the Zn–BLD fillers. An ee value of 74% for R-methyl phenyl sulfoxide (R-MPS) over S-MPS was obtained, and this ee value was higher than that of the pure Zn–BLD membrane prepared via a reactive seeding method on a zinc oxide support (ee: 33%) [28]. 

Recently, an ee value of 100% was successfully achieved using MIL–53–NH–*L–His* incorporated mixed matrix membranes [62]. MIL–53–NH nanocrystals were synthesized, and a post-synthetic modification was performed, grafting a chiral amino acid (*L*–Histidine) into the frameworks, creating homochiral MIL–53–NH–*L–His* fillers. The filler nanocrystals were dispersed in a polyethersulfone (PES) solution following the reported protocol to prepare a solid-additive-included polymer solution [35,63,64]. The mixture was cast into a flat sheet film using a casting blade. The filler loading was varied from 10 to 30 wt%, and the highest ee value of 100% for R–(+)–phenylethanol over S–(–)–phenylethanol was obtained with 20 wt% loadings. The number of chiral recognition sites was low with 10 wt% loadings. It was found that 30 wt% loadings caused partial aggregation of the fillers, which could create more defects, lowering the quality of the enantioselective membranes. The ee value of 100% was valid during the initial stage of permeation: the dominant transport mechanism of this MMM was a facilitated transport, which inevitably involves non-enantioselective diffusion through the polymeric matrix, lowering the separation performance for long-term operation [16]. The parameters such as the concentration of enantiomeric feed solution, membrane thickness, and the loading amount of chiral fillers should be carefully designed to enhance the performance of the enantioselective MMMs.

**Figure 3:**
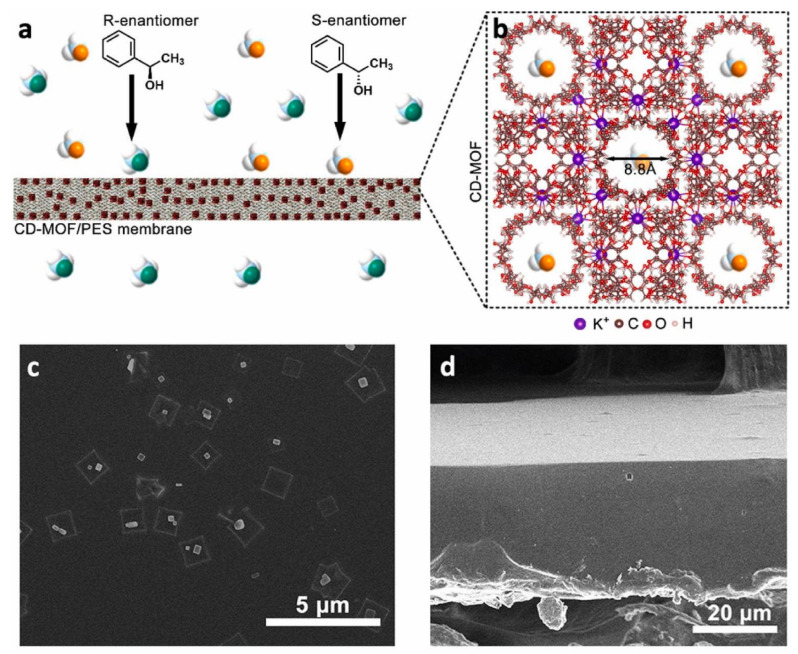
Schematic illustration and SEM images of γ–cyclodextrins–based metal–organic frameworks (CD–MOF)/polyethersulfone mixed matrix membranes and (**a**) CD–MOF/PES MMM for selective transport of R–(+)–1–phenylethanol molecules from racemic mixture. (**b**) 3D structure of CD–MOF. (**c**) SEM image of CD–MOF/PES MMM surface and (**d**) cross–section. Reprinted with permission from [42] Copyright 2021 Membrane Science.

Another homochiral MOF was incorporated into a well-designed MMM with good membrane quality. γ –cyclodextrins-based MOFs (γ–CD–MOF) have been known to separate a wide variety of enantiomeric mixtures. This ability is attributed to the 40 stereogenic centers present in each γ-CD torus [65]. The γ–CD–MOF based MMM (Figure 3) was prepared by adopting a procedure similar to preparing MIL-53-NH-L-His/PES: the mixture of γ–CD–MOF and PES solution was cast into a self-standing membrane [42]. The ee value of 100% for R–(+)–1–phenylethanol over S–(–)–1–phenylethanol was obtained with a non-polar solvent of n-hexane. However, because the polar solvent (e.g., methanol, ethanol) preferentially occupies the active sites of cyclodextrins [49] and destroys hydrogen bonding between the 1-phenylethanol and γ–CD–MOF [65], the enantioselective adsorptive performance of γ–CD–MOF/PES with a polar solvent was relatively worse. The optimized loading amount of γ–CD–MOF fillers in MMM was 20 wt%, similar to MIL–53–NH–L–His/PES. This work revealed that the polarity of solvents used for the enantiomer separation should be chosen carefully considering the chemical stability of the chiral filler in enantioselective MMMs. 

### 3.2. Chiral Filler: Covalent Organic Frameworks (COFs)

Covalent organic frameworks (COFs) are porous organic materials formed by strong covalent bonding of organic building units, thus creating highly porous materials [46]. Its pore structure and pore size can be tuned by various ligands based on coordination chemistry similar to the synthesis of MOFs. COFs have distinct features of high surface area and tailored functionality, which are directly related to their intriguing structural advantages in the fields of adsorption, catalysts, and gas storage applications [46,66,67,68]. Like chiral MOFs, COFs built by chiral ligands have chirality within the COFs structures. Interestingly, COFs composed of an achiral ligand can also exhibit chirality, which can be induced during the synthesis procedure. Han et al. reported a unique strategy to prepare chiral COFs (CCOFs) with achiral linkers via chiral catalyst-induced immobilization. The CCOFs were synthesized using 1,3,5–triformylphloroglucinol (Tp) with an achiral diamine or triamine linkers in the presence of catalytic amounts of (R)–or (S)–1–phenylethylamine. The chirality of the produced COFs was confirmed by circular dichroism (CD) spectroscopy [69]. Most COFs are difficult to use as filler material in HPLC or mixed matrix membrane due to their broad size distribution after synthesis. The non-uniform particle sizes can result in a high degree of packing density in the column. It can result in a large pressure drop and eddy diffusion issues, which are not suitable for packed bed operation [70]. As a solution, Ma et al. provided size-controllable synthesis by adjusting the amount of catalyst (acetic acid, HAc) under acetonitrile (ACN) solvent. The experimental results confirmed that uniform larger particles were generated as the amount of acetic acid was reduced to 0.7 and 0.3, respectively [71].

**Figure 4:**
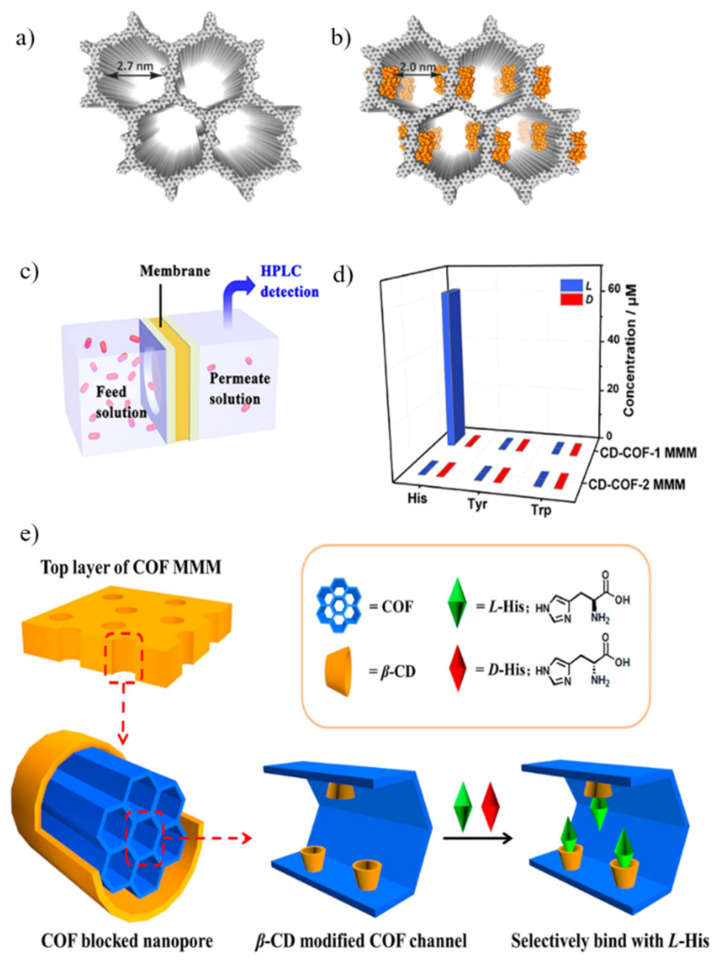
(**a**) Top view of covalent organic framework–1 (COF–1). (**b**) Chemically modified COF-1 with β–cyclodextrin (β–CD). (**c**) Scheme of penetrating amino acids via membrane. (**d**) Concentration of permeate solution after transporting. (**e**) Schematic illustration of chiral CD–COF MMM system. Reproduced with permission from [74] Copyright 2019 American Chemical Society.

Since chiral COFs have the potential to separate enantiomers based on their chiralities [69,70,72], it is reasonable to expect that COFs can be utilized as filler particles in enantioselective mixed matrix membranes [70]. The limitations of chiral COFs remain in the high cost and difficulty of production of chiral monomers. However, such restrictions can be overcome with post-synthetic modification of the COF nanochannels [73]. Yuan et al. synthesized COF–1 and COF–2 via solvothermal reactions of 1,3,5–tris(4–formylphenyl)benzene, 2,5–divinylbenzene–1,4–diamine, and *p*–phenylenediamine (for COF–1) or *o*–tolidine (for COF–2) with different 2D packing parameters of AA (COF–1) and AB stacking (COF–2). AA stacking is the preferred configuration, since it has a single type of pore structure that can be effectively modified by *β*-cyclodextrin (β–CD). Subsequently, the nanochannels of COFs were decorated with *β*-CD via thiol-ene click reactions, and the CD–COF–1 demonstrated a selective binding affinity to *L*–histidine (Figure 4) [74].

The post-synthetic modification of COF nanochannels can also be performed using biomolecules. Zhang et al. performed post–synthetic modifications of COF–1, introducing biomolecules such as amino acids, peptides, and enzymes that have chiralities. Nanochannels anchored by biomolecules possess strong covalent bonds, resulting in a stable structure. Zhang et al. reported that the enantioselectivity depends on the structural complexity, the number of chiral centers, and the amphipathicity of the biomolecules. Considering the amphiphilicity and zwitterionic features of biomolecules, it is expected that biomolecule-modified COFs will have enantioselectivity. Thus, biomolecule-modified COFs are another type of chiral fillers that can be incorporated into enantioselective MMMs [73].

#### Dispersed Chiral COF Fillers in an Achiral Polymeric Matrix 

The tunable, isolated pore channel of COF can be converted into a chiral environment by introducing chiral selectors. Chiral COF–7 (CCOF–7) was synthesized using a chiral linker of 6,6′–dichloro–2,2′–diethoxy–1,1′–binaphthyl–4,4′–dialdehyde, and it was dispersed in a PVDF solution containing DMF and acetone as a mixed solvent [72]. Electrospinning of the CCOF–7/PVDF MMM was performed with various CCOF–7 loadings, from 5 to 10 wt%. This work did not present the results of enantiomer separation; however, it was confirmed that free-standing MMMs composed of a chiral COF in a polymeric matrix could be successfully fabricated via electrospinning as a proof-of-concept. CD-COF–1/PES MMM was the first report to develop a COF–based enantioselective MMM [74]. The post-synthetic modification of COF-1 via thiol-ene click reaction between COF-1 and 6–deoxy–6–mercapto–*β*–cyclodextrin (*β*–CD) was performed to prepare a chiral CD-COF–1. A CD–COF–1 and polyethersulfone suspension in DMF was cast on the glass to form MMM by phase inversion. The free-standing CD–COF–1/PES achieved a high separation factor (*L*–histidine/*D*–histidine) of ~34.0 and suggested that a COF-based MMM could be a versatile platform, with pore-tunability for the selective transport of small molecules, including an enantiomeric mixture. Table 1 summarizes the separation performance of the reported MMMs based on MOFs or COFs.

## 4. Other Composite Membranes 

### 4.1. Filler: Zeolites

**Figure 5:**
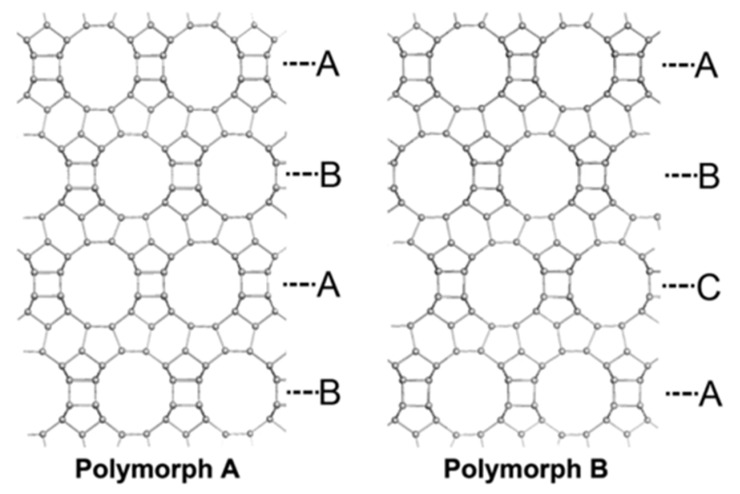
Top view of polymorph A and polymorph B, showing their distinctive stacking order. Reprinted with permission from [79] Copyright 2015 Scientific Reports.

In addition to the fillers mentioned above, other materials can also have potential as candidate materials for MMM. Zeolites are porous inorganic frameworks that have been broadly utilized in the petrochemical industry as catalysts and adsorbents [75,76]. Zeolites can be classified into various space groups depending on their structures [77]. Because of its structural advantages of well-defined pore structures and 3D-interconnected structures, zeolites can adsorb specific molecules smaller than their pore size. Since zeolites integrate shape selectivity, chiral zeolites have been extensively explored in the field of enantiomer separation. Among the many zeolite groups, groups with intrinsic chirality built from chiral units can be grouped: BEA, CZP, GOO, ITV, JRY, LTJ, OSO, and STW [78]. Similar to the helical structure of chiral MOFs, chiral zeolites also have a right- or left-handed configuration [79]. For example, HPM–1, which possesses helical pores, is one of the STW–type zeolites with intrinsic chirality [80]. Another chiral zeolite group, beta zeolites (BEA), also have a helical structure. Three distinct polymorphs–A, –B, and –C are stacked while forming the zeolite beta structure (Figure 5). In polymorph–A, only right-handed or left-handed orientation layers are piled, resulting in a pure chiral helical channel. Like the previously mentioned HPM–1, the pure polymorph-A form also has an enantioselective pore [78,81]. 

**Figure 6:**
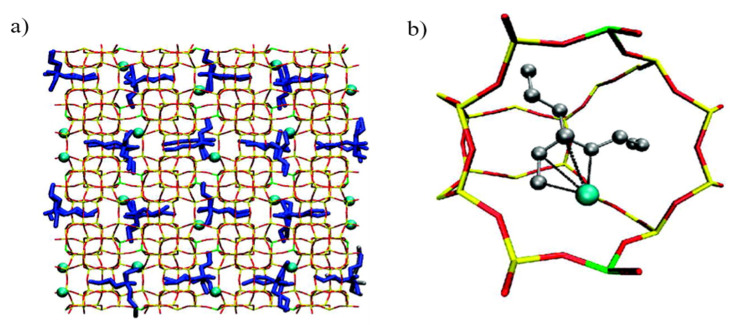
(**a**) Adsorption arrangement of 4–ethyl–4–methyloctane molecules (S/R gas ratio = 7:3) in zigzag and straight channel with Al-5(Ca^2+^). S/R-enantiomers are colored blue and gray, respectively. The blue spheres indicate Ca^2+^ cations. (**b**) Magnified image of enantioselective adsorption at a specific intersection; the colors represent oxygen (red), silicon (red), and aluminum (green) Reprinted with permission from [85] Copyright 2010 American Chemical Society.

Another characteristic of zeolites is that zeolites have a negative framework charge, attributed to the Si/Al ratio. Caused by a negative charge on the framework, cations such as Na^+^ are present in the pores. As a consequence, zeolites are often characterized as ion-exchange materials [82]. Using the ‘ion exchange’ property, there are also zeolites with enantioselectivity, despite their achiral structure. Van Erp et al. discovered that the zeolite MFI, which has 12 different silicon positions, could exhibit chirality even though the MFI channel is a simple achiral zigzag channel. According to their results, when substituted with attached Al, Ca ions, the orientation varied depending on where they were attached, which results in enantioselectivity. The Si/Al ratio is essential to separate the chiral molecules, giving the structure a specific orientation [83]. The ability to resolve enantiomers was also proven by Monte Carlo Modeling, which supported the previous results [84] (Figure 6). Other interactions should be considered when incorporating zeolites into MMMs. The interface structure should be regarded to ensure a defect-free composite membrane [85], which can depend on interactions between the inorganic material, zeolite, and organic membrane. Poor adhesion between the zeolite and polymer can form undesirable channels, degrading the selectivity of the MMM. That is, the sieve–in–a–cage structure could be formed, which results in non-selective bypassing of the target molecule [86]. 

### 4.2. Filler: Porous Organic Cages (POCs)

Among the new class of porous materials, porous organic cages (POCs) with shape-persistent, three-dimensional porous molecules with accessible cavities have received significant attention for chiral resolution. CC3 cages, produced by reactions between 1,3,5–triformylbenzene (TFB) with (1R,2R)–1,2–cyclohexanediamine (CHDA), in particular, have been of great interest for analytical chiral resolution. Because the CC3–family has the characteristics of shape selectivity and size selectivity like MOFs, it can perform well in enantiomer separation. In particular, an HPLC column prepared with CC3–R (chirally pure CC3 in R–configuration) showed better performance than a commercial column in separating chiral alcohols and diols. Besides this case, there are plenty of enantiomers such as 2–butylamine, 2–chlorobutane, 1–methoxy–2–hydroxypropane that could be resolved in a CC3–R column [87]. 

Generally, the conventional synthesis protocols of POCs have had limitations: long reaction times and complex catalysts/solvents were inevitable. However, one of the eco-friendly synthetic routes for CC3–R has been reported to not require toxic catalysts during its preparation. Wang et al. discovered a catalyst-free ethanolic refluxing synthesis, and the product of CC3–R enabled gas chromatography (GC) based enantiomeric alcohol separation. GC columns packed with CC3–R showed higher selectivity for most chiral alcohols than commercial columns, *β*–DEX 225, and Cyclosil-B columns [88]. 

Given its advantages, the CC3–family is suitable for various enantiomer separation applications. CC3–R has a high surface area (>1000 m^2^/g), excellent enantioselectivity, and thermal/chemical stability, and therefore, it is a suitable candidate for fillers in enantioselective MMMs. A CC3–R–incorporated polyvinyl chloride (PVC) membrane electrode enabled a separation of isomers of 2-amino–1–butanol. CC3–R was stable in a membrane electrode even after being treated with tetrahydrofuran or rinsed with water for 48 h, implying stability under organic solvents. The constituents of the membrane, the loading amount of CC3–R, or the pH of the environment affected its enantioselectivity, and the maximum efficiency of the CC3–R–PVC membrane electrode was achieved at a pH of 9.0 [89]. In addition to the CC3–family, CC10, one of the imine-linked POCs, also has enantiomer separation ability. CC10 combined with a polysiloxane OV–1701 could be packed into the chiral column for gas chromatography. CC10 showed better enantioselectivity to chiral alcohols, esters, ketones, ethers, halo-hydrocarbons, epoxides, and organic acids compared to the commercial approach. Moreover, because CC10 has excellent potential for separating *n*-alkanes, *n*-alcohols, Grob mixture, and positional isomers, CC10 is a promising candidate material for practical application [90]. 

**Figure 7:**
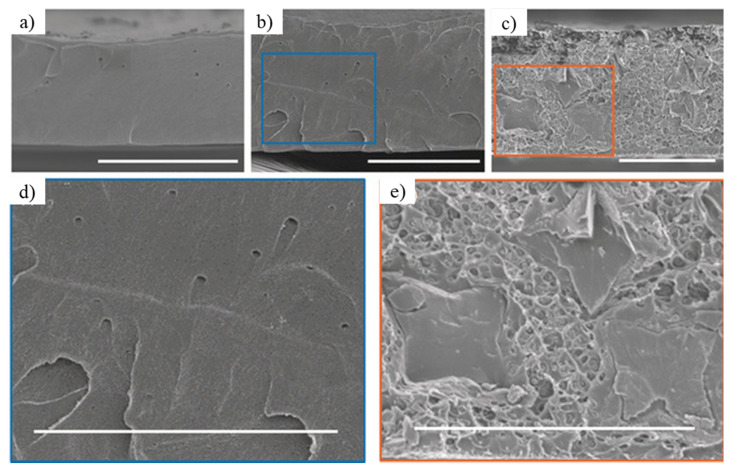
SEM image of a molecularly mixed matrix membrane, that shows CC3–R crystals within the Matrimid matrix [92]. (**a**) Pristine polymer Matrimid (**b**) 10 wt % porous organic cage (POC), (**c**) 20 wt % POC, (**d**,**e**) magnified image. Reproduced with permission from [93]. Copyright 2019 Angewandte Chemie International Edition.

The most attractive feature of POCs is their high solubility in organic solvents, which enables convenient and straightforward solution processing with polymers [91]. POCs can be easily incorporated into ‘molecularly’ mixed composite membranes (MMCMs, Figure 7) because of their solubility. POCs show a higher degree of dispersion than other filler materials in MMM (e.g., MOF, COF, zeolite, etc.) because the molecular cages can interact with a polymeric matrix at the molecular level. These composite membranes show better performance in both permeability and selectivity for gas separation [91]. Moreover, a composite membrane of mixed CC3–R and polymers with intrinsic microporosity (PIM–1) could enhance both permeability and aging resistance. Because POCs can be dissolved in a polymer solution and mixed into a polymeric matrix, the persistent issue of fabricating defect-free membranes at a large scale can be resolved [92,93]; thus, enantioselective MMM or MMCM can be developed with POCs.

### 4.3. Other Composite Mixed Matrix Membrane

**Figure 8:**
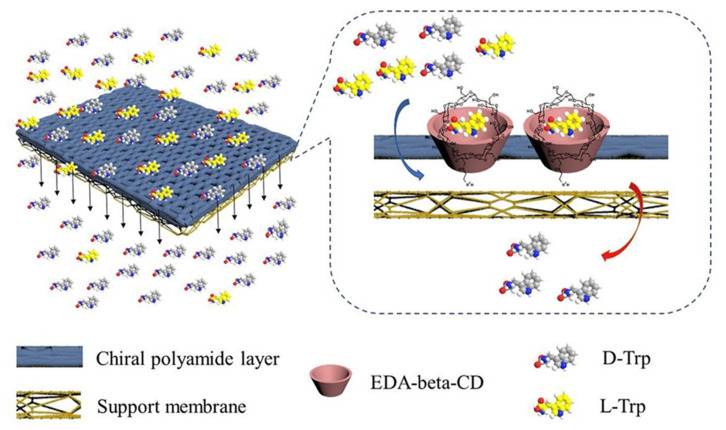
Illustrations of molecular transport mechanisms in the membranes. Reprinted with permission from [44] Copyright 2020 Membrane Science.

Besides the porous materials such as zeolite and POCs, chiral molecular complexes or chiral functionalized carbon materials have been explored in mixed matrix membrane architecture. (Table 2) The chiral EDA–β–CD–TMC/cellulose acetate mixed matrix membrane was fabricated using in-situ interfacial polymerization (Figure 8) [44]. 

The obtained ee values were 27.2% for the D–/L–tryptophan mixture, 9.29% for the (±)-warfarin mixture, and 3.77% for the (±)-ibuprofen mixture. The calculated complex formation energy of EDA-β-CD-L-tryptophan (∆G_L_: −58 kcal/mol) was lower than that of EDA–β–CD–D–tryptophan (∆G_D_: −54 kcal/mol) [94]. Therefore, L–tryptophan was retained in the membrane forming the EDA–β–CD–L–tryptophan complex, and D–tryptophan diffused through the polymeric membrane phase, showing the retarded transport mechanism. Single-walled carbon nanotubes (SWCNT) were functionalized using D–tryptophan and a homogeneous mixture of polysulfone (PSf), polyethylene glycol (as an additive), and the functionalized SWCNTs in NMP were cast on a glass plate [43]. The SWCNT/PSf was prepared via phase inversion and showed an ee value of 98.86% for L-tyrosine over D-tyrosine. Due to the selective binding of the D–tyrosine, the flux of L-tyrosine was much higher, and this implies that this enantioselective MMM is a sorption-selective membrane.

The typical MMM consists of a polymeric matrix that can stably hold microporous filler particles. However, suppose we extend the range of MMM to other classes of ‘mixed’ matrices. In that case, some membranes with discrete chiral selectors can be classified as enantioselective MMMs. A protonated graphite carbon nitride (GCN)–based membrane was tuned by incorporating (1R)–(-)–10–camphorsulfonic anion (CSA) [95]. A GCN–CSA suspension was turned into a membrane via vacuum filtration on a mixed cellulose ester or polytetrafluoroethylene (PTFE) substrate. The precise control of the interlayer spacing of the membrane enabled enantioselective permeation, resulting in a high ee value of 89% for (+)–limonene over (-)–limonene. The molecular weight cutoff was around 150 g/mol; thus, enantiomeric mixtures with a molecular weight higher than 150 g/mol (e.g., carvone, phenylalanine, tryptophan, etc.) cannot be separated with this membrane.

Interestingly, forms opposite to the typical MMMs can also be considered. An achiral graphene oxide (GO) nanosheet-based membrane was converted to an enantioselective MMM via integration with chiral polymers. The GO nanosheets were treated with L–glutamic acid (L–Glu), and the chiral polymer (Poly–L–glutamate, PLGA) filled the interlayer spacing of Glu–GO nanosheets to control the flux and induce the chirality [96]. Glu-GO/PLGA film was fabricated on a cellulose acetate (CA) membrane, and the enantioselectivity of this mixed matrix membrane toward 4–Dihydroxy–D–phenylalanine (D–DOPA)/L–DOPA (α(D–DOPA)⁄(L–DOPA)) was 2.8, which was an improvement compared to the Glu–GO membrane (α(D–DOPA)⁄(L–DOPA) was around 2.0) [97]. Following this work, various GO–based enantioselective membranes have been reported. A modified Glu–GO by bulky carboxyl-terminated ionic liquid (IL–COOH) showed improvement in both flux and separation factor (α(D–DOPA)⁄(L–DOPA) = 3.83) due to the increased channel spacing [98]. Another type of GO membrane modified by L–phenylalanine was also reported [99]. Combining the GO membrane’s high permeability with a chiral polymer filler’s enantioselectivity could be one of the new designs of enantioselective MMMs.

## 5. Prospects for Designing Enantioselective MMMs

Most reported enantioselective MMMs are diffusion–selective membranes with facilitated transport mechanisms; thus, the major challenge for these membranes is the reduction of enantioselectivities over time. As non-enantioselective diffusion of the enantiomer causes this issue, reducing the diffusion rate of the other enantiomer would be a good option for maintaining high enantioselectivity for an extended period of operation. To achieve this, we can imagine two possible scenarios: (1) finding a suitable polymer with a smaller and more rigid intrinsic microporosity; (2) changing the membrane fabrication process to obtain a denser polymeric matrix. As the permeability and selectivity are inversely proportional to each other, lowering the diffusion rate of non-enantioselective species (i.e., decreasing the permeability) can enhance the enantioselectivity, affecting the overall chiral resolution. Therefore, for large-scale MMM-based enantiomeric separation processes, the polymeric matrix should be carefully designed to find the optimized separation condition. As mentioned in Section 3, a cyclodextrin (CD)-MOF-based MMM and COF-based MMM were recently developed, and they achieved approximately 100% of ee value over 24 and 36 h, respectively. Such diffusion-selective MMMs could be one of the plausible options for continuous enantiomeric separation [42,74]. Various CD-based MOFs or COFs can be synthesized via reticular chemistry; thus, a new class of MOFs or COFs can be prepared for use as chiral fillers to develop versatile enantioselective MMMs.

Most of the enantioselective MMMs in Section 3 were prepared using an achiral polymeric matrix (i.e., the polymeric matrix does not have enantiomeric separation ability itself). A few chiral polymers with a high ee value (e.g., (+)–PIM–CN or (+)–PIM–COOH [26], an optically pure form of a polymer of intrinsic microporosity) have been reported, suggesting we can design a proper configuration of chirality in both the filler and the polymeric matrix of the MMM to enhance the enantioselective performance. As discussed earlier, chiral (P)-Eu(BTC) was incorporated into the achiral PIM-1 matrix to fabricate an enantioselective MMM, but the highest ee value was only 9% for the 2-amino-1-butanol [60]. If chiral (+)–PIM–CN or (+)–PIM–COOH was adopted as a polymeric matrix, the enantiomeric separation ability of the MMM could be improved due to the increased affinity of a chiral PIM to (+)–2–amino–1–butanol. Another reported enantioselective MMM (MIL–53–NH–L–His/polyethersulfone) exhibited an ee value of 100% for R–(+)–1–phenylethanol over S–(-)–1–phenylethanol [62]. As the ee value was high enough, this MMM appears to be a promising candidate for enantioselective MMM-based chiral resolution. However, the ee value decreased to 59% after eight hours due to lower stability. In this case, we could imagine that utilizing the chiral (+)–PIM–CN or (+)–PIM–COOH matrix would preserve the high ee value much longer; however, this type of enantioselective MMM is still a diffusion-selective membrane. Since the critical factor in improving stability is delaying the non–enantioselective diffusion of S–(-)–1–phenylethanol, controlling the affinity or pore size of the polymeric matrix could maintain a higher ee value for long-term operation. A modified diffusion-selective membrane with chiral polymers would solve this issue; it could be practically utilized in the delicate separation of a low-concentration enantiomeric mixture.

Regardless of the transport mechanism, we also need to focus on the structure of MMM, which consists of a polymeric matrix and nano-sized filler particles. First, as the polymeric matrix is typically unstable to the solvent, we need to find a way to improve the chemical stability of the membrane. The vapor phase infiltration (VPI) technique was recently adopted to create intertwined metal oxide networks within a PIM–1 structure [33]. The VPI–treated PIM–1 membrane was not swollen or dissolved by an aggressive solvent; thus, this VPI treatment can be integrated with a chiral PIM–1–based enantioselective MMM [26] as well as in organic solvent reverse osmosis applications [33]. Next, when the nano–sized filler particles are incorporated into the polymeric matrix, the interaction between discrete filler particles and the polymeric matrix should be controlled to avoid bypass formation. Various strategies have been suggested to increase interfacial compatibility and improve the performance of MMMs. ‘Priming’ is a traditional strategy to prevent filler–filler interaction. A layer of polymer adsorbed onto the surface of the filler particle could hinder this filler–filler interaction [35]. Recently, a post-synthetic modification of UiO–66–NH_2_ was reported: the surface of the UiO–66–NH_2_ was functionalized with 4,4′–(hexafluoroisopropylidene)diphthalic anhydride (6FDA)-Durene oligomers via amine-anhydride reaction to increase the interfacial compatibility between the MOF and polymer. This modified MOF was successfully incorporated into defect-free 6FDA–Durene MMMs, which subsequently showed enhanced gas permeability and selectivity [100]. These strategies would be useful in fabricating enantioselective MMMs with minimum effects of sieve–in-a–cage morphology or defects. Porous organic cages (POCs) and metal-organic polyhedra (MOPs) have been used as fillers in one phase of ‘molecularly’ mixed composite membranes (MMCMs), and the MMCM did not suffer from the poor interface issue [101]. As various types of chiral POCs are reported [91,102], enantioselective MMCMs can be developed in the future. There is a growing demand in industries involved with the energy-efficient production of enantiomerically pure compounds for pharmaceuticals and food additives. Membranes with advanced performance and structure will provide scalable options for ultimate enantiomeric separation at scale.

## Figures and Tables

**Table 1:** MOF- or COF-based mixed matrix membranes.

Polymer	Filler	Loading Amount (wt%)	Target Material	ee	Ref.
PIM-1	Eu(BTC)	30	2–amino–1–butanol	9%	[60]
HDPE	Zn–BLD	86	MPS	74%	[28]
PES	MIL–53–NH–L–His	20	phenylethanol	100%	[62]
PES	γ–CD–MOF	20	phenylethanol	100%	[42]
PVDF	CCOF–7	5–10	-		[72]
PES	CD–COF–1	-	D–/L–Histidine	34 (α_(D⁄L))	[74]

**Table 2:** Other composite mixed matrix membranes.

Composite 1	Composite 2	Target Material	ee	Ref.
CA	EDA–*β*–CD	D–/L–tryptophan	27.2%	[44]
CA	EDA–*β*–CD	(±)–warfarin	9.29%	[44]
CA	EDA–*β*–CD	(±)–ibuprofen	3.77%	[44]
PSf	SWCNT	D–/L–tyrosine	98.86%	[43]
mixed cellulose ester or PTFE	GCN–CSA	(±)–limonene	89%	[95]
CA	Glu–GO/PLGA	4–Dihydroxy–D–phenylalanine	2.8(α_(D⁄L))	[96]

## Data Availability

No new data were created or analyzed in this study. Data sharing is not applicable to this article.
